# Alterations in gut microbiota composition in adult patients with familial Mediterranean fever: a pilot case-control study

**DOI:** 10.55730/1300-0144.6214

**Published:** 2026-02-25

**Authors:** Nazife Şule YAŞAR BİLGE, Vicente PEREZ BROCAL, Timuçin KAŞİFOĞLU, Uğur BİLGE, Nilgün KAŞİFOĞLU, Andres MOYA, Ener Çağrı DİNLEYİCİ

**Affiliations:** 1 Division of Rheumatology, Department of Internal Medicine, Faculty of Medicine, Eskişehir Osmangazi University, Eskişehir, Turkiye; 2 Area of Genomics and Health, Foundation for the Promotion of Health and Biomedical Research of Valencia Region (FISABIO-Public Health), Valencia, Spain; 3 Biomedical Research Networking Center for Epidemiology and Public Health (CIBEResp), Madrid, Spain; 4 Department of Family Medicine, Faculty of Medicine, Eskişehir Osmangazi University, Eskişehir, Turkiye; 5 Department of Microbiology, Faculty of Medicine, Eskişehir Osmangazi University, Eskişehir, Turkiye; 6 Department of Pediatrics, Faculty of Medicine, Eskişehir Osmangazi University, Eskişehir, Turkiye

**Keywords:** Familial Mediterranean fever, FMF, microbiota, *Eggerthella lenta*

## Abstract

**Background/aim:**

Although familial Mediterranean fever (FMF) is a monogenic disease, recent evidence suggests that the gut microbiota may play a role in its pathogenesis or phenotypic expression. We conducted a pilot exploratory study to evaluate the intestinal microbiota composition in patients with FMF and compare it to that of healthy controls.

**Materials and methods:**

In this prospective cohort study, 10 adult patients with FMF receiving colchicine and 10 age- and sex-matched healthy controls were enrolled. Fecal samples were collected and stored at −80 °C until DNA extraction. The V3–V4 region of the 16S rRNA gene was amplified and sequencing was performed.

**Results:**

Alpha and beta diversity metrics were largely similar between FMF patients and the control group, except for the Chao 1 index, which was significantly reduced in the FMF group (p < 0.05), indicating lower species richness. Taxonomic analysis revealed differences in gut microbiota composition, notably an increased abundance of *Eggerthella* at the genus level. At the species level, *Eggerthella sinensis* and *Eggerthella lenta* were more prevalent in FMF patients.

**Conclusion:**

Our findings reveal distinct gut microbiota alterations in FMF patients, characterized by reduced microbial richness and particularly enrichment of *Eggerthella*, including *E. lenta*. These findings suggest a possible association between gut microbiota alterations and FMF. The small sample size of this study is a limitation, but further longitudinal studies with treatment-naïve patients, alongside functional analyses of microbial metabolites, are warranted to elucidate causal relationships and inform microbiome-based diagnostic or therapeutic strategies in FMF.

## 1. Introduction

Familial Mediterranean fever (FMF) is the most common monogenic autoinflammatory disease and predominantly affects populations originating from the Mediterranean basin, including individuals of Turkish, Armenian, Arab, and Jewish descent [1]. The disease is characterized by recurrent self-limited episodes of fever, serositis, and elevated inflammatory markers [2]. FMF is caused by mutations in the *MEFV* gene, which encodes pyrin, a regulatory protein of the innate immune system involved in inflammasome activation and inflammatory signaling [3].

Despite its monogenic nature, FMF exhibits marked heterogeneity in clinical manifestations, disease severity, treatment response, and the development of complications such as AA amyloidosis [1,4]. Pyrin senses alterations in host cellular processes induced by bacterial activity rather than recognizing specific pathogen-associated molecular patterns, suggesting that host–microbiota interactions may influence inflammatory responses in FMF [5]. Alterations in gut and oral microbiota composition have been implicated in the pathogenesis of several autoinflammatory and autoimmune diseases [6,7].

To date, only a limited number of studies have investigated the gut microbiota composition in FMF patients, and most of these studies focused on pediatric populations or patients with severe disease phenotypes [8–10]. Previous studies have reported reduced microbial diversity and enrichment of proinflammatory taxa, particularly in patients with colchicine resistance or AA amyloidosis [8,9]. However, results remain inconsistent, likely due to differences in study design, cohort characteristics, and analytical approaches. Notably, data regarding gut microbiota composition in adult FMF patients are lacking.

Therefore, the aim of this study was to characterize the fecal microbiota profile of adult FMF patients and compare it with that of age- and sex-matched healthy controls, with a particular focus on taxonomic differences at the genus and species levels.

## 2. Materials and methods

### 2.1. Study design and participants

The larger Rheuma-BIOTA study is an observational study designed to investigate the intestinal microbiota composition of patients with rheumatological diseases [10]. The present analysis was conducted in the Department of Rheumatology of the Eskişehir Osmangazi University Faculty of Medicine, focusing on patients diagnosed with FMF.

This prospective cohort study included 10 adult FMF patients diagnosed according to the Tel Hashomer criteria [11]. A control group consisting of 10 age- and sex-matched healthy individuals was recruited using strict inclusion and exclusion criteria. Patients were included if they were receiving no medications other than colchicine. All patients were in remission and clinically stable at the time of sampling. All were receiving a stable dose of colchicine for at least 6 months prior to sample collection. Age- and sex-matched healthy adults were recruited as controls.

Exclusion criteria for both the FMF group and the control group were as follows: age of <18 years; smoking habit; use of antibiotics or probiotics within the previous 8 weeks; body mass index of >30 kg/m^2^; chronic medical conditions including diabetes mellitus, cardiovascular disease, inflammatory bowel disease (ulcerative colitis or Crohn’s disease), functional bowel disorders, prior gastrointestinal surgery, or malignancy; and acute gastrointestinal infection or symptoms requiring medical treatment.

All procedures were performed in accordance with the ethical standards of the relevant institutional and/or national research committee and the principles of the Declaration of Helsinki and its later amendments. Written informed consent was obtained from all participants.

### 2.2. Clinical data collection

Demographic and clinical data were obtained through retrospective review of medical records. Collected variables included sex, age at the time of study enrollment, age at the time of symptom onset, age at diagnosis, clinical manifestations (fever, peritonitis, pleuritis, erysipelas-like erythema, myalgia, arthritis, and sacroiliitis), disease-related complications (amyloidosis and chronic renal failure), and colchicine dosage.

### 2.3. Genetic analysis

*MEFV* mutation analysis results were retrieved from patients’ medical records when available.

### 2.4. Stool sample collection and DNA extraction

All participants provided a minimum of 5 mL of fresh stool sample. Samples were collected in sterile Falcon tubes and immediately stored upright at −80 °C until DNA extraction. Total genomic DNA was extracted from weighed fecal aliquots using the QIAamp DNA Stool Mini Kit (QIAGEN, Hilden, Germany) according to the manufacturer’s instructions. Extraction controls were included throughout the procedure. Extracted DNA samples were quantified and subsequently shipped on dry ice for sequencing and downstream analysis.

### 2.5. 16S rRNA gene amplification, library preparation, and sequencing

The V3–V4 hypervariable regions of the 16S rRNA gene were amplified following the Illumina 16S Metagenomic Sequencing Library Preparation protocol (Illumina, San Diego, CA, USA) and amplicon primers were selected. Polymerase chain reaction (PCR) amplification was performed using 12.5 ng of genomic DNA per sample under the following conditions: initial denaturation at 95 °C for 3 min, followed by 25 cycles of denaturation at 95 °C for 30 s, annealing at 55 °C for 30 s, and extension at 72 °C for 30 s. Amplicons were visualized on 1.4% agarose gels and quantified using a Qubit 3.0 fluorometer (Thermo Fisher Scientific, Waltham, MA, USA).

Indexing was performed using the Nextera XT Index Kit (Illumina) by adding dual indices (N7xx and S5xx) in a second PCR (8 cycles, identical thermal profile). Indexed libraries were pooled in equimolar concentrations and sequenced on the MiSeq platform using the MiSeq Reagent Kit v3 (2 × 300-bp paired-end reads; Illumina). Negative PCR controls, extraction controls, and internal sequencing controls were included throughout the workflow.

### 2.5. Bioinformatics and statistical analysis

Raw paired-end sequencing reads were subjected to quality control using the publicly available PRINSEQ-lite (v0.20.4) tool with the following parameters: min_length: 50; trim_qual_right: 30; trim_qual_type: mean; trim_qual_window: 20.

Filtered reads were processed using the DADA2 pipeline, which included quality filtering and trimming, the learning of error rates, dereplication, amplicon sequence variant inference, paired-end merging (minimum of 15-bp overlap), and chimera detection and removal.

To eliminate potential host contamination, reads were mapped against the human reference genome (GRCh38.p11) using Bowtie2 (v2.3.4.2) with the “very sensitive” option. Unmapped reads were retained for downstream analysis. Taxonomic assignment was performed using a naïve Bayesian classifier against the SILVA reference database, complemented by BLASTn confirmation when required. Downstream ecological and compositional analyses were performed with QIIME 2 (v2018.6.0) and R software.

Alpha diversity was assessed using the Chao 1 richness estimator and Shannon diversity index. Rarefaction was performed (1000 iterations at 20,000 reads per sample). Group comparisons were conducted using nonparametric tests with Monte Carlo permutations.

Beta diversity was evaluated using Bray–Curtis dissimilarity and binary Jaccard distances. Principal coordinate analysis was used for visualization. The statistical significance of group separation was assessed using PERMANOVA (Adonis). Differentially abundant taxa between FMF patients and the control group were identified using linear discriminant analysis effect size (LEfSe) via the Galaxy platform. The linear discriminant analysis score threshold was >2.0 and statistical significance was accepted at p < 0.05. Demographic data were presented as mean ± standard deviation. Due to the small sample size, formal subgroup and multivariate analyses were not performed.

## 3. Results

### 3.1. Clinical and genetic characteristics of FMF patients

The study included 10 FMF patients (8 women and 2 men) with a mean age of 33.3 ± 10.24 years and a mean disease duration of 17.1 ± 10.32 years. All patients had a history of fever and peritonitis. Additional clinical manifestations included erysipelas-like erythema (n = 7), pleuritis (n = 6), arthritis (n = 4), and sacroiliitis (n = 1). All patients were receiving colchicine therapy and none were treated with interleukin-1 inhibitors.

Genetic analysis was available for nine patients: five were homozygous for the M694V mutation, one was heterozygous for M694V, one had compound heterozygous M694V/R761H, one had M694V/E148Q, and one was homozygous for M680I.

### 3.2. Gut microbiota composition

#### 3.2.1. Microbial diversity

##### 3.2.1.1. Shannon index

**Figure 1:**
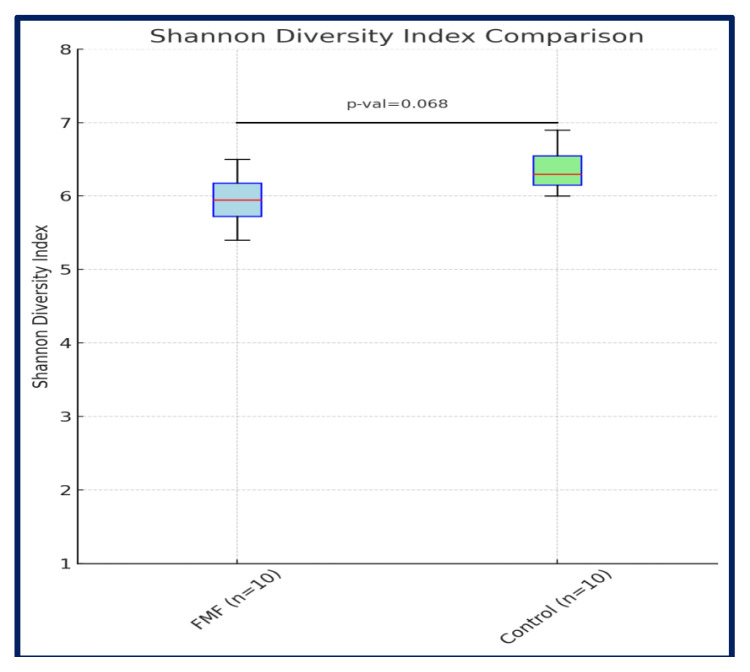
Boxplot of Shannon diversity index in familial Mediterranean fever (FMF) patients and healthy controls. Comparison of the Shannon diversity index between FMF patients (n = 10) and healthy controls (n = 10). The box represents the interquartile range, the horizontal line indicates the median, and whiskers represent minimum and maximum values.

The FMF patients showed a trend toward reduced microbial diversity compared to the healthy controls; however, this difference did not reach statistical significance (p = 0.068) (Figure 1).

##### 3.2.1.2. Chao 1 index

**Figure 2:**
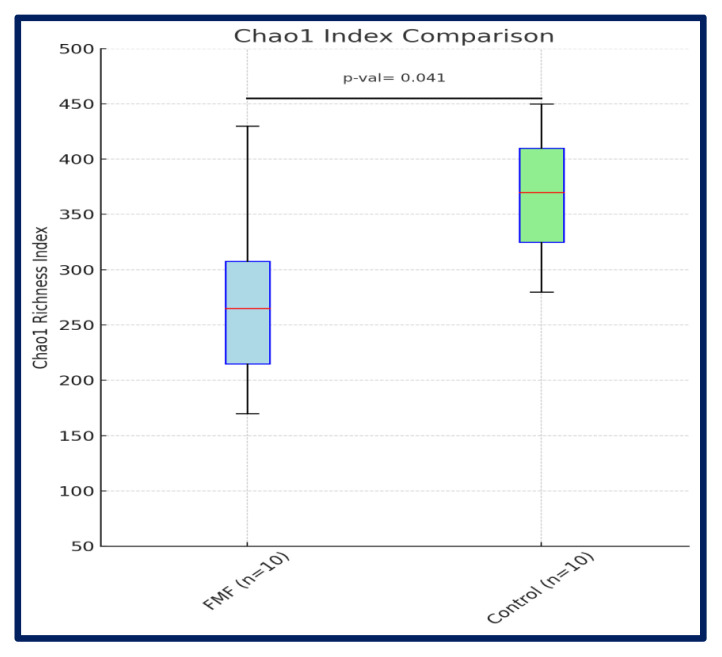
Boxplot of Chao 1 richness index in familial Mediterranean fever (FMF) patients and healthy controls. Comparison of the Chao 1 index between FMF patients (n = 10) and healthy controls (n = 10). The box represents the interquartile range, the horizontal line indicates the median, and whiskers represent minimum and maximum values.

The Chao 1 richness index was significantly lower in FMF patients than in healthy controls (p = 0.041), indicating reduced microbial richness in the FMF cohort (Figure 2).

#### 3.2.2. Taxonomic composition

**Figure 3:**
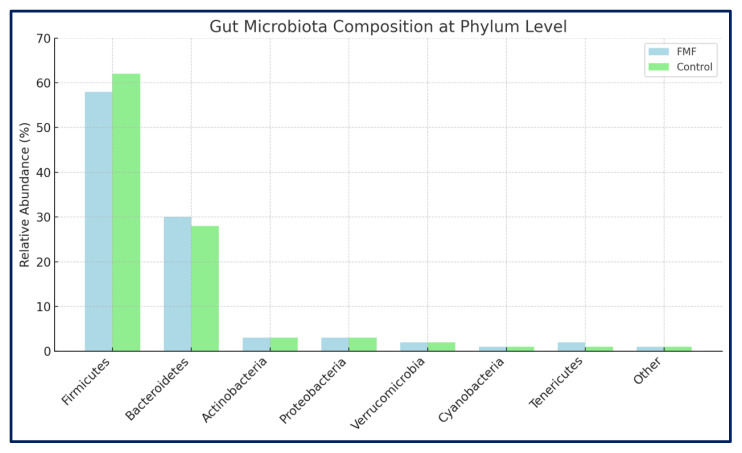
Gut microbiota composition at the phylum level in familial Mediterranean fever (FMF) patients and healthy controls. The bar plot shows the relative abundance (%) of dominant bacterial phyla. Firmicutes and Bacteroidetes were the most abundant phyla in both groups, with a relatively higher proportion of Firmicutes in controls compared to FMF patients.

At the phylum level, Firmicutes and Bacteroidetes predominated in both groups. Firmicutes accounted for 59.5% of the microbiota in FMF patients and 62.3% in the control group, whereas Bacteroidetes accounted for 31.5% and 27.9%, respectively. Consequently, the Firmicutes/Bacteroidetes ratio was lower in FMF patients (1.88) than in the control group (2.23) (Figure 3).

**Figure 4:**
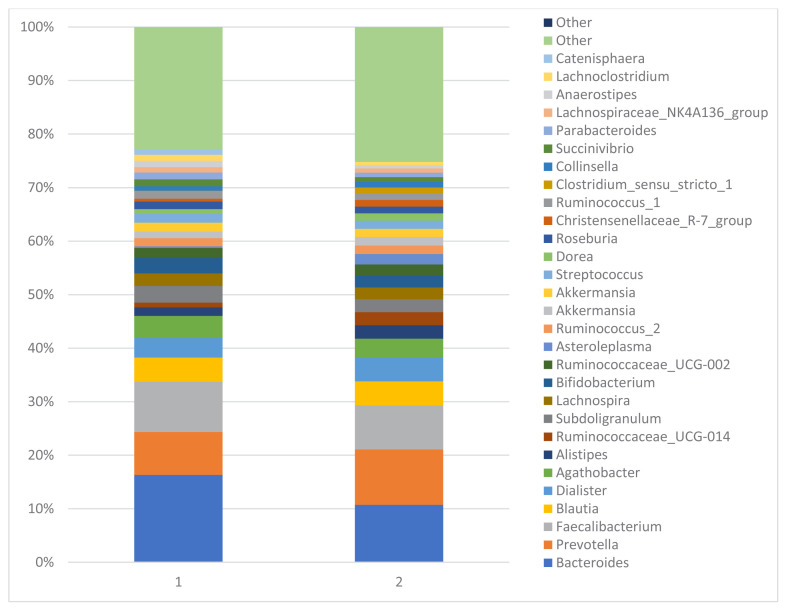
Stacked bar plot representing the relative abundance of bacterial genera in familial Mediterranean fever patients (1) and healthy controls (2). The x-axis indicates the study groups, while the y-axis represents the proportion of each bacterial genus relative to the total microbiota. The color-coded segments represent the most abundant genera identified, including *Bacteroides*, *Prevotella*, *Faecalibacterium*, *Blautia*, and others.

At the genus level, FMF patients exhibited reduced relative abundance of beneficial taxa such as *Faecalibacterium*, *Roseburia*, and the Christensenellaceae R-7 group, while *Collinsella*, *Clostridium* sensu stricto 1, and *Succinivibrio* were relatively enriched (Figure 4). At the species level, *Eggerthella lenta* and *Eggerthella sinensis* were more abundant in FMF patients.

**Figure 5:**
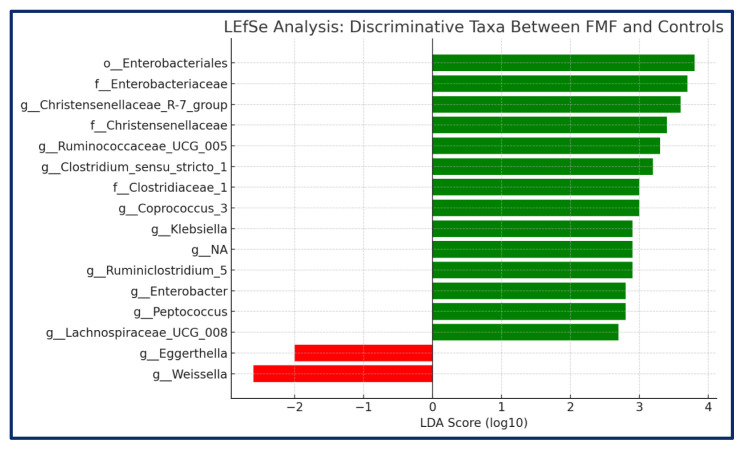
Linear discriminant analysis effect size (LEfSe) analysis identifying discriminative bacterial taxa between familial Mediterranean fever (FMF) patients and healthy controls. The x-axis represents the linear discriminant analysis (LDA) score (log_10_), reflecting the effect size of each taxon in differentiating the groups. Green bars indicate taxa enriched in FMF patients, while red bars represent taxa enriched in healthy controls. o: Order; f: family; g: genus.

LEfSe analysis identified the *Christensenellaceae R-7* group, *Ruminococcaceae UCG_005*, *Coprococcus* 3, and *Clostridium* sensu stricto 1 as significantly enriched in the control group, whereas *Weissella* and *Eggerthella* species were enriched in FMF patients (Figure 5).

## 4. Discussion

This study provides novel data on gut microbiota composition in adult FMF patients. We observed a significant reduction in microbial richness, as reflected by the Chao 1 index, in FMF patients compared to healthy controls. Although Firmicutes and Bacteroidetes remained the dominant phyla in both groups, the reduced Firmicutes/Bacteroidetes ratio in FMF patients may reflect a shift in microbial composition, with early dysbiosis associated with chronic inflammation [12].

At the genus level, reductions in butyrate-producing and antiinflammatory taxa, particularly *Faecalibacterium*, were observed [13]. Conversely, enrichment of potentially proinflammatory genera such as *Eggerthella* was also detected [14]. Notably, *E. lenta* and *E. sinensis* were more abundant in FMF patients. These species have been associated with mucosal immune activation and chronic inflammatory conditions [14,15]. Experimental studies have demonstrated that *E. lenta* can promote Th17-mediated immune responses and systemic inflammation, supporting its potential relevance in FMF pathophysiology [16]. The increased abundance of *E. lenta* has also been reported in several immune-mediated diseases, including inflammatory bowel disease, rheumatoid arthritis, multiple sclerosis, and multisystem inflammatory syndrome in children [17].

Our findings are consistent with previous studies reporting altered microbiota composition in FMF, particularly in patients with severe disease phenotypes [8,9]. Differences between pediatric and adult cohorts may reflect age-related microbiota dynamics, environmental factors, and long-term colchicine exposure [18]. Additionally, bile acid-mediated pyrin inflammasome activation [19] and host genetic factors [20,21] may further modulate host–microbiota interactions in FMF.

The primary limitation of this study is the small sample size, which limits the statistical power and increases the risk of type II error. Another important limitation is the inability to clearly distinguish disease-specific microbiota alterations from treatment-related or environmental influences. Given the cross-sectional design and the inclusion of only colchicine-treated patients in remission, the observed microbial differences may reflect a composite effect of chronic disease, long-term therapy, and host–environment interactions. Therefore, causality cannot be inferred and our findings should be interpreted as exploratory. Longitudinal studies including newly diagnosed treatment-naïve FMF patients are needed to identify microbiota changes directly attributable to disease pathogenesis. Given the limited sample size, the present study should be considered a pilot exploratory analysis aimed at identifying potential microbiota signatures in adult FMF patients.

In conclusion, adult FMF patients exhibit reduced microbial richness and distinct taxonomic alterations compared to healthy controls. The enrichment of *Eggerthella*, particularly *E. lenta*, may represent a microbial signature associated with autoinflammation in FMF. These findings suggest a potential association between gut microbiota composition and FMF, warranting further investigation in larger adequately powered studies.
